# Selecting Tyrosine Kinase Inhibitors for Gastrointestinal Stromal Tumor with Secondary KIT Activation-Loop Domain Mutations

**DOI:** 10.1371/journal.pone.0065762

**Published:** 2013-06-20

**Authors:** Yuan-Shuo Hsueh, Chih-Lung Lin, Nai-Jung Chiang, Chueh-Chuan Yen, Chien-Feng Li, Yan-Shen Shan, Ching-Huai Ko, Neng-Yao Shih, Ling-Mei Wang, Ting-Shou Chen, Li-Tzong Chen

**Affiliations:** 1 National Institute of Cancer Research, National Health Research Institutes, Tainan, Taiwan; 2 Institute of Clinical Pharmacy and Pharmaceutical Science, National Cheng Kung University, Tainan, Taiwan; 3 Biomedical Technology and Device Research Laboratories, Industrial Technology Research Institute, Hsinchu, Taiwan; 4 Department of Internal Medicine, National Cheng Kung University Hospital, Tainan, Taiwan; 5 Division of Hematology and Oncology, Department of Medicine, Taipei Veterans General Hospital, Taipei, Taiwan; 6 National Yang-Ming University School of Medicine, Taipei, Taiwan; 7 Department of Pathology, Chi-Mei Foundation Medical Center, Tainan, Taiwan; 8 Division of General Surgery, Department of Surgery, National Cheng Kung University Hospital, Tainan, Taiwan; 9 Department of Internal Medicine and Cancer Center, Kaohsiung Medical University Hospital, Kaohsiung Medical University, Kaohsiung, Taiwan; National Cancer Centre, Singapore

## Abstract

Advanced gastrointestinal stromal tumors (GIST), a *KIT* oncogene-driven tumor, on imatinib mesylate (IM) treatment may develop secondary *KIT* mutations to confer IM-resistant phenotype. Second-line sunitinib malate (SU) therapy is largely ineffective for IM-resistant GISTs with secondary exon 17 (activation-loop domain) mutations. We established an *in vitro* cell-based platform consisting of a series of COS-1 cells expressing *KIT* cDNA constructs encoding common primary±secondary mutations observed in GISTs, to compare the activity of several commercially available tyrosine kinase inhibitors on inhibiting the phosphorylation of mutant KIT proteins at their clinically achievable plasma steady-state concentration (Css). The inhibitory efficacies on *KIT* exon 11/17 mutants were further validated by growth inhibition assay on GIST48 cells, and underlying molecular-structure mechanisms were investigated by molecular modeling. Our results showed that SU more effectively inhibited mutant KIT with secondary exon 13 or 14 mutations than those with secondary exon 17 mutations, as clinically indicated. On contrary, at individual Css, nilotinib and sorafenib more profoundly inhibited the phosphorylation of KIT with secondary exon 17 mutations and the growth of GIST48 cells than IM, SU, and dasatinib. Molecular modeling analysis showed fragment deletion of exon 11 and point mutation on exon 17 would lead to a shift of KIT conformational equilibrium toward active form, for which nilotinib and sorafenib bound more stably than IM and SU. In current preclinical study, nilotinib and sorafenib are more active in IM-resistant GISTs with secondary exon 17 mutation than SU that deserve further clinical investigation.

## Introduction

Gastrointestinal stromal tumors (GISTs) are the most common type of mesenchymal tumors in the gastrointestinal tract and usually refractory to cytotoxic chemotherapy and radiotherapy. Recently, 85–90% of GISTs are found to harbor gain-of-function mutations of KIT or platelet-derived growth factor receptor (PDGFR), which leads to promoting cell proliferation and escaping from apoptosis [1]. Over 90% of primary *KIT* mutations in GIST occur in either juxtamembrane domain (exon 11) or extracellular domain (exon 9), and rarely in the cytoplasmic ATP-binding domain (exon 13/14) or activation-loop domain (exon 17) [2].

**Figure 1:**
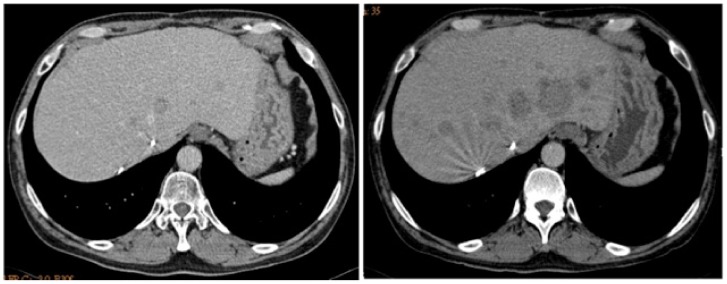
Rapid progression of IM-resistant tumor after SU treatment. A patient harboring KIT exon 11^Val555_Leu576del^/17^Asn822Lys^ mutated, metastatic GIST within the liver after 3 months of SU at dose of 50 mg/day, 4 weeks-on/2 weeks-off., (a) before and (n) after SU treatment.

Imatinib mesylate (IM; Gleevec®, Novartis Pharma, Basel, Switzerland) and sunitinib malate (SU; Sutent®, Pfizer Inc., USA) are oral multiple tyrosine kinase inhibitors (TKIs) competing with ATP for the ATP-binding site of several receptor tyrosine kinase. Both of them selectively block the activation of KIT and PDGFR [3]. Currently, IM 400 mg/day is the standard first-line treatment for unresectable or metastatic, non-exon 9 *KIT* mutated GISTs and 800 mg/day for exon 9 mutated ones, with a clinical benefit response rate and median progression-free survival (PFS) of 85% and 2.3 to 4.0 years, respectively [4]. The well recognized mechanisms of IM resistance include acquired an add-on secondary mutation on the ATP binding domain or the activation-loop domain of KIT, overexpression of KIT, loss of KIT expression accompanied with activation of alternative pathways, TKI-induced quiescence, or potential role of cancer stem-cells [5]. Among them, acquired secondary *KIT* mutation is the most commonly observed etiology [5], [6]. Based on the results of two clinical trials, the current standard of care for IM-refractory GISTs is SU [7], [8]. However, genotype analysis showed that patients with secondary *KIT* mutation involving activation-loop domain have poor PFS and overall survival (OS) [7], [9]. In nowadays, SU remains the standard of care for IM-refractory GISTs regardless the status of their secondary *KIT* mutation. Clinically, some patients with secondary *KIT* mutation involving activation-loop domain experienced rapid disease after switch their treatment from IM to SU, as shown in Fig. 1.

In the past few years, several commercially available TKIs, for example, nilotinib, dasatinib and sorafenib, are under clinical investigation for IM/SU-resistant GISTs. Nilotinib is designed based on the structure of IM and shows higher affinity to the ATP-binding site of ABL kinase to overcome IM-resistant chronic myeloid leukemia (CML) and also selectively inhibits KIT and PDGFR [10]. Dasatinib, an oral TKI for both BCR-ABL and Src family, is also a second-line drug for patients with IM-resistant CML and able to inhibit the activation of exon 11^Val560Asp^ or exon 17^Asp816Val^ KIT mutants [11]. Sorafenib is a multi-target inhibitor actively against BRAF, vascular endothelial growth factor receptor 2/3, PDGFR, and KIT^Trp557_Lys558del/Thr670Ile^ mutant expressed in Ba/F3 system and also has the activity to suppress the growth of GIST with KIT exon 11 fragment deletion in xenograft mouse model [12]–[14]. Besides, recent data suggested that all three agents exhibit varied degree of activity against IM/SU-resistant GISTs with a disease-control rate (DCR, complete or partial response plus stable disease for 6 months or more) ranging from 25 to 70%. Unfortunately, genotype study has rarely been reported in these clinical studies. Whether individual KIT TKI preferntially against certain genotype of IM/SU-resistant GISTs remain largely unknown. This issue is clinically important because it may not only be useful in selecting optimal treatment for IM/SU-resistant GISTs but also help to identify potentially more active second-line treatment for IM-resistant GISTs with secondary activation-loop mutation, for which SU showed little activity.

In this study, we used an *in vitro* cell-based drug screening platform, which consists of a series of COS-1 cells expressing *KIT* cDNA constructs encoding mutant exon 9^Ala502_Tyr503insAlaTyr^, 11^Val555_Leu576del^, 11^Val560Asp^, 13^Val654Ala^, 14^Thr670Ile^, 17^Asp820Gly^, and 17^Asn822Lys^ either alone or in combination to mimicking the common *KIT* mutations observed in GISTs, to study the potential activity of several commercially available KIT TKIs at their achievable serum steady-state concentration. The results were further validated by their growth inhibition activities on human GIST48 cells with exon 11/17 double mutations. In addition, we applied molecular modeling/docking analysis to delineate the underlying molecular structure mechanisms of interaction between TKIs to mutant KIT proteins.

## Materials and Methods

### Construction of *KIT* Mutants

A 2.9 kB full length complimentary DNA (cDNA) of wild-type *KIT* was obtained by reverse transcription PCR from the messenger RNA (mRNA) isolated from peripheral blood mononuclear cells of a volunteered study initiator, cloned into *pcDNA3.1/Zeo* (Invitrogen, Carlsbad, CA), and confirmed by sequencing. *KIT* mutants with single and/or double mutations were constructed using QuickChange site-directed mutagenesis kit (Stratagene, La Jolla, CA), while one mutant with long segment deletion of exon 11 from 555 to 576 (exon 11^Val555_Leu576del^) was attained using slicing overlap extension PCR [15]. The primers were listed in Table S1. The signed informed consent was obtained from the blood donor and the recombinant DNA experiment was approved by Human Ethics Committee and Institutional Review Board, National Health Research Institutes, Taiwan.

### Cell Lines and Reagents

COS-1 cells were obtained from Dr. Shih (Neng-Yao laboratory, National Health Research Institute, Taiwan) where they acquired from The NHRI Cell Bank and maintained in 10% FBS/DMEM (Hyclone, Waltham, MA). GIST48 cells, with a homozygous exon 11^Val560Asp^ and a heterozygous exon 17^ Asp820Ala^ mutation, were a gift from Fletcher (Harvard Medical School, Boston, MA) and maintained in 15% FBS/F10 (Invitrogen) plus 1% bovine pituitary extract and 0.5% Mito+™ serum extender (BD Biosciences, San Jose, CA) as previous reports [5], [9]. IM and nilotinib, and sorafenib were kindly supplied by Novartis and Bayer, respectively; while SU and dasatinib were purchased commercially. Primary antibodies for total KIT and KIT^Y703^ were purchased from DAKO (Düsseldorf, Germany) and Invitrogen, respectively. Other primary antibodies included ACTIN (Millipore, Billerica, MA), AKT, AKT^S473^ (Cell Signaling Technology, Danvers, MA), and horseradish peroxidase (HRP) labeled secondary antibodies (Jackson ImmunoResearch Laboratories, West Grove, PA).

### Transient Transfection and TKI Treatment

Transfection was performed using Lipofectamine 2000® according to manufacture’s protocol (Invitrogen). In brief, COS-1 cells at about 90% confluence in 6-well dishes were admixed with 2 µg of plasmid *KIT/pcDNA3.1* and 2 µL of Lipofectamine 2000® for 6 hours. Transfected cells were recovered by incubation in growth media for 18 hours. After another 2-hour starvation in FBS-free DMEM, the transfected cells were treated with TKIs for 30 minutes before harvested. GIST48 cells were treated with TKIs for 30 minutes after starved 2 hours in F10 medium without serum.

### Immunoblotting Studies

Cells were lysed in CelLytic™ M reagent (Sigma-Aldrich, St. Louis, MO) containing protease and phosphatase inhibitors. Protein concentration was determined by Bradford method (Bio-Rad, Hercules, CA). Equal protein of each sample was separated by SDS-PAGE and transferred to PVDF membrane. After blocking and incubating with primary antibody and then with HRP labeled secondary antibody, immunostains were detected by enhanced chemiluminescence (ECL), developed by autoradiography and quantified using the 1Dscan Ex gel analysis software (Scanalytics, Rockville, MD). The activation ratio of KIT after TKI treatment was estimated by comparing the densitometry ratio of phospho-KIT/total KIT bands and plotting the percentage of activation relative to untreated control. Considering the diversification of TKIs’ achievable concentrations in clinical, we compared the inhibitory effects of TKIs on KIT phosphorylation at individual steady-state concentration (Css), and expressed as inhibitory ratio (1 - activation ratio) at Css (IR_Css_). The Css of IM, SU, nilotinib, dasatinib, and sorafenib at their regular clinical dosing are determined as 1000 (at 400 mg/day), 200 (at 50 mg/day), 1000 (at 400 mg/day), 40 (at 200 mg/day), or 4750 (at 800 mg/day) nM, respectively following the literatures [16]–[20]. Data was expressed as mean ± S.E.

### Growth Inhibition Assay

1×10^4^ GIST48 cells were seeded in each well of 24-well plates and then exposed to TKIs for 72 h. The methylene blue dye assay was used to evaluate the relative number of viable cells after TKIs treatment, measured at O.D. 595, and normalized to the DMSO-only control group. The cell viabilities were determined after plotting the percentage of growth relative to untreated control. The survival ratio at the clinically achievable Css of each TKI was estimated from plotted relative cell viabilities. Data was expressed as mean ± S.E.

### Molecular Modeling and Docking

Biopolymer module of SYBYL-X (Tripos, St. Louis, MO) was used to introduce single and double mutations into the wild-type structure. Five known KIT crystal structures from Protein Data Bank library were selected as templates (PDB id: 1PKG, 1T45, 1T46, 3G0E, and 3G0F). 1PKG was used as the template for fully active KIT and others as templates for inactive KIT to build the corresponding models for individual TKIs. The mutant models were charged with Gästeiger-Hückel method and minimized using the Amber force field (version 7.0 or FF99) with a steepest descent gradient by a conjugate gradient of 0.025 kcal/mol or a maximum of 50000 iterations as termination criteria [21], [22]. Docking TKIs to the binding site of simulation model was performed by program module Glide in Schrödinger Suite 2011 (Schrödinger, LLC). The GlideScore function in SP mode was used in all docking stages, and the binding energy was deduced from the GlideScore function.

## Results

### Effects of TKIs on KIT with Single Mutation Expressed in COS-1 Cells

We constructed series *KIT* mutants containing single or double mutations. Single mutations were those commonly observed in clinical samples, including exon 9^Ala502_Tyr503insAlaTyr^, exon 11^Val560Asp^, 13^Val654Ala^, 14^Thr670Ile^, and 17^Asp820Gly and Asn822Lys^. In addition, one mutant containing frequent deletion region, exon 11^Val555_Leu576del^, was also constructed to investigate whether mutants with a substitution or a long segment deletion of exon 11 would respond differently to TKIs. Double mutations were generated a secondary mutant (exon 13^Val654Ala^, 14^Thr670Ile^, 17^Asp820Gly^, and 17^Asn822Lys^) on primary mutant (exon 9^Ala502_Tyr503insAlaTyr^, 11^Val555_Leu576del^, exon 11^Val560Asp^), respectively. COS-1 cells expressed these constructs were incubated with each TKI and then analyzed KIT phosphorylation by immunoblotting or luminex assay. IM and SU were firstly used to validate the correlation between the findings from our screening platform and currently clinical data. Other commercially available TKIs, including nilotinib, dasatinib, and sorafenib, were evaluated their inhibitory effects on IM- and/or SU-resistant mutants.

**Figure 2:**
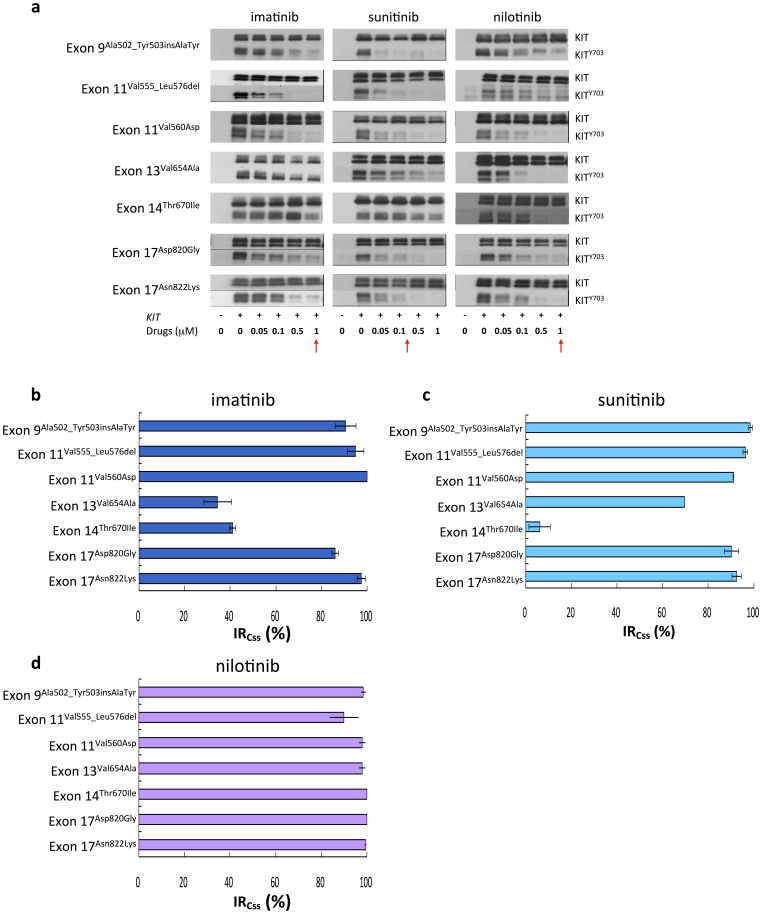
Effects of TKIs on phosphorylated KIT with variant KIT mutations. (a) COS-1 cells transfected with KIT single mutants were starved and treated with indicated doses of IM, SU, and nilotinib for 30 minutes. The total expression and degree of phosphorylation of KIT were determined by western blot analysis. (b) Inhibitory ratios at Css (IR_Css_) of IM, SU, and nilotinib, as the red arrow pointed, on KIT single mutations were determined by quantification of phosphorylated KIT/total KIT and estimated from the western blot in (a). The data are expressed as the mean ± SE of three independent experiments.

IM, SU, and nilotinib could inhibit the phosphorylation of mutant KIT proteins with single exon 9^Ala502_Tyr503insAlaTyr^, exon 11^Val555_Leu576del^, and exon 11^Val560Asp^ mutation in COS-1 cells (Fig. 2a-d). IM also inhibited KIT phosphorylation of exon 17^Asp820Gly^ and exon 17^Asn822Lys^ mutants, but totally ineffective for exon 13^Val654Ala^ and exon 14^Thr670Ile^ mutants. SU, as IM, could inhibit the phosphorylation of exon 17^Asp820Gly^ and exon 17^Asn822Lys^ mutants, but only partially effective and totally ineffective for exon 13^Val654Ala^ and exon 14^Thr670Ile^ mutants, respectively. On the other hand, nilotinib effectively inhibited all four mutants. Moreover, the effects of imatinib on *KIT* mutants of exon 9^Ala502_Tyr503insAlaTyr^, exon 11^Val555_Leu576del^, and exon 11^Val560Asp^ from luminex assay and immunoblotting were markedly compatible, as Figure S1 shown. Beyond these validations, these results strengthen the reliability of our *in vitro* cell-based platform.

### Effects of TKIs on KIT Mutants with Secondary ATP-binding Domain Mutations

**Figure 3:**
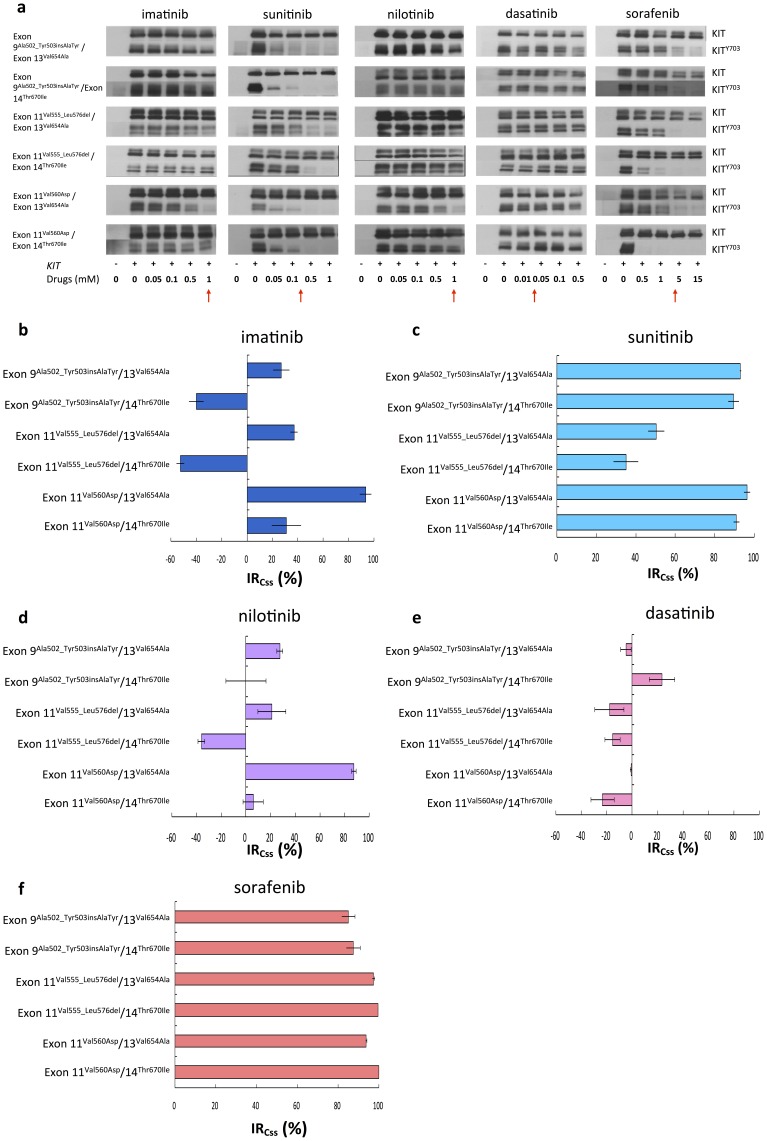
Effects of TKIs on phosphorylated KIT with mutations on exon 9 or 11/13 or 14. (a) COS-1 cell transfected with KIT double mutants were starved and treated with indicated doses of multiple TKIs for 30 minutes respectively. The total expression and degree of phosphorylation of KIT were determined by western blot analysis. (b) Inhibitory ratios at Css (IR_Css_) of multiple TKIs, as the red arrow pointed, on KIT secondary mutations on exon 13 or 14 were determined by quantification of phosphorylated KIT/total KIT and estimated from the western blot in (a). The data are expressed as the mean ± SE of three independent experiments.

We further examined the inhibitory efficacies of TKIs against KIT phosphorylation harboring secondary ATP-binding domain (exon 13 or 14) mutations. Compatible to previous reports, IM was not effective for mutants with secondary exon 13 or 14 mutations (Fig. 3a and 3b). In contrast, SU and sorafenib effectively inhibited KIT phosphorylation of these mutants (Fig. 3a, 3c, and 3f). Nilotinib worked on mutants of exon 11^Val560Asp^/13^Val654Ala^ but totally failed on other secondary exon 13/14 mutants (Fig. 3d). Dasatinib was largely ineffective to inhibit the phosphorylation of KIT with secondary exon 13 or 14 mutations (Fig. 3e). Based on our data, SU had better inhibitory effect on KIT mutants with exon 9 or 11/13 or 14 double mutations than IM, nilotinib, and dasatinib that is consistent with the observation in SU phase III trial and demonstrates that our *in vitro* cell-based platform is reliable. Moreover, sorafenib showed similar inhibitory effect on these double mutants as SU, even could have better effects on exon 11^Val555_Leu576del^/13^Val654Ala^ and exon 11^Val555_Leu576del^/14^Thr670Ile^ mutants.

### Effects of TKIs on KIT Mutants with Secondary Activation-loop Domain Mutations

**Figure 4:**
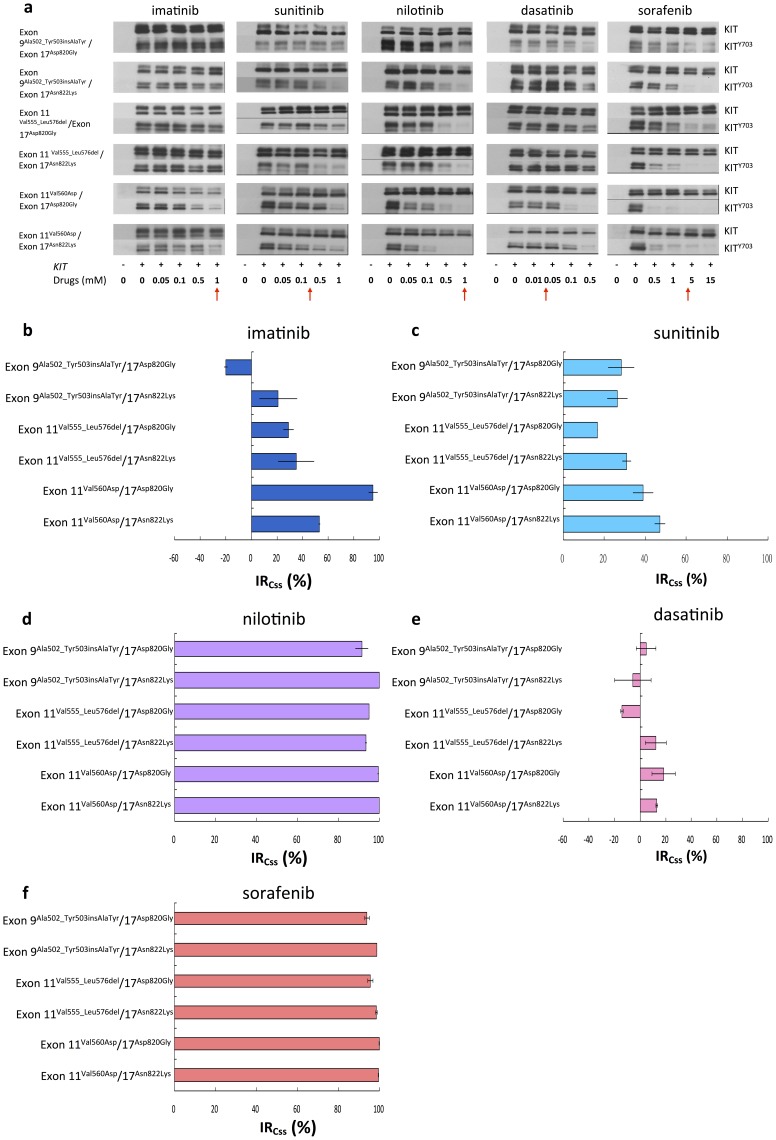
Effects of TKIs on phosphorylated KIT with mutations on exon 9 or 11/17. (a) COS-1 cell transfected with KIT double mutants were starved and treated with indicated doses of multiple TKIs for 30 minutes respectively. The total expression and degree of phosphorylation of KIT were determined by western blot analysis. (b) Inhibitory ratios at Css (IR_Css_) of multiple TKIs, as the red arrow pointed, on KIT secondary mutations on exon 17 were determined by quantification of phosphorylated KIT/total KIT and estimated from the western blot in (a). The data are expressed as the mean ± SE of three independent experiments.

**Figure 5:**
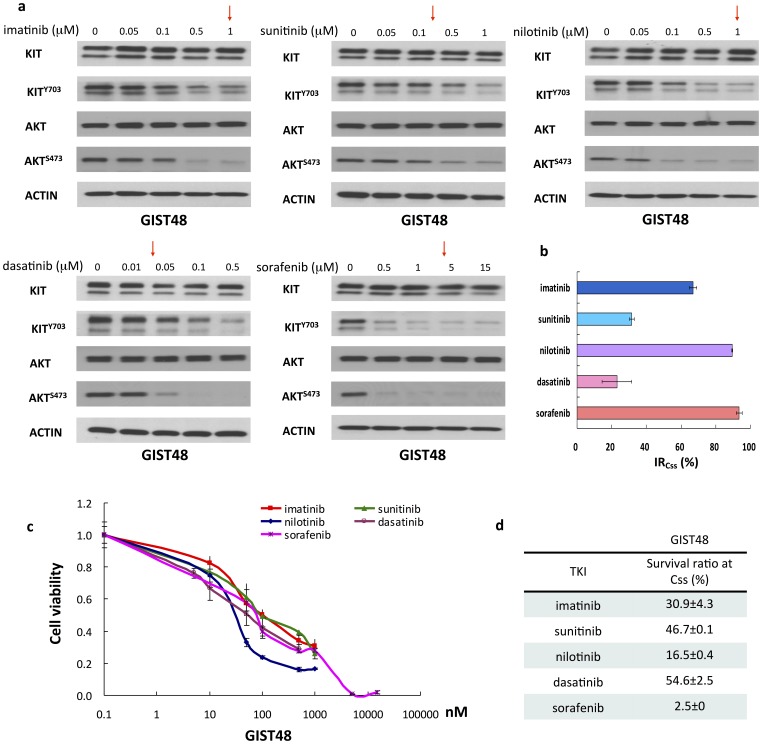
Effects and antitumor activities of TKI on GIST48 cells. (a) GIST48 cells were starved and treated with indicated doses of TKIs for 30 minutes respectively. The total expression and degree of phosphorylation of KIT were determined by western blot analysis. (b) Inhibitory ratios at Css (IR_Css_) of TKIs, as the red arrow pointed, on mutated KIT of GIST48 were determined by quantification of phosphorylated KIT/total KIT and estimated from the western blot in (a). (c) Antitumor activities of TKIs against GIST48 cells were preformed with TKIs as indicated doses for 3 days respectively. The cell viabilities were determined by comparing each data to untreated control. (d) Survival ratios at Css of TKIs against GIST48 were estimated from (c). The data are expressed as the mean ± SE of three independent experiments.

On secondary activation-loop domain (exon 17) mutants, nilotinib had superior inhibitory activity on all 9 or 11/17 mutants than SU (Figs. 4a and 4d). Dasatinib had minor or no inhibitory effect against all exon 9 or 11/17 mutants (Figs. 4a and 4e). Interestingly, sorafenib was actively against the phosphorylation of KIT mutants with secondary exon 17 mutations (Figs. 4a and 4f). Furthermore, GIST48 cells were used to validate the inhibitory efficacies of TKIs whether led to KIT signal cascades downregulation and cell death. Consistent with mutant KIT expressing COS-1 model, at the concentration of their Css, nilotinib and sorafenib showed more potent inhibitory efficacies toward KIT phosphorylation, AKT activation, and cell growth on GIST48 cells than IM, SU, and dasatinib (Figs. 5a–d).

### Virtual Molecular Modeling/docking Analysis

**Figure 6:**
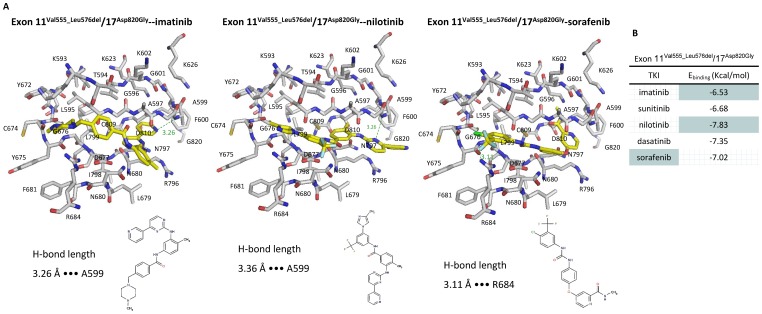
Binding energies and interactions of TKIs to KIT 11^Val555_Leu576del^/17^Asp820Gly^ mutations. (a) Stereo views of IM, nilotinib, and sorafenib binding to KIT showing key hydrogen bonds formed with A599 and R684 in different models. (b) Binding energies of IM, SU, nilotinib, dasatinib, and sorafenib were estimated according docking TKIs to KIT mutants on exon 11^Val555_Leu576del^/exon 17^Asp820Gly^.

Based on our *in vitro* studies, we found that the differential response of doubly mutated KIT to various TKIs. Therefore, molecular modeling of mutated KIT and TKIs docking were used to elucidate the interactions between KIT mutant proteins and TKIs. Previous molecular modeling indicated that single mutations on exon 9, 11, and 13 would cause instability of auto-inhibited KIT conformation and shift the equilibrium toward active conformation. However, a comprehensive molecular modeling for TKIs specificities toward IM-resistant double mutated KIT is rarely described. So we established the molecular model of KIT exon 11^Val555_Leu576del^/17^Asp820Gly^ mutant protein as previous described and trained it using 100 kinase inhibitors. The simulation model showed that combination of a segmental deletion of exon 11 and point mutation on exon 17 resulted in a shift of KIT kinase from inactive form close to fully active form. The dockings of five TKIs onto the ATP-binding site of KIT mutant protein were showed as Fig. 6a and Fig. S2. The binding energies of TKIs to exon 11^Val555_Leu576del^/17^Asp820Gly^ indicated that nilotinib was the most potent inhibitors for exon 11/17 double mutants (Fig. 6b).

## Discussion

In current study, we used an *in vitro KIT* expressing COS-1 cell-based system to evaluate the inhibitory efficacy of several commercial available TKIs on phosphorylation of KIT with secondary mutation involving exon 17, and validated the findings on their growth inhibition activity on GIST 48 cells harboring exon 11/17 KIT mutant. In contrast to other *in vitro* studies, we included a long-segmental exon 11 deletion mutation, exon 11^Val555_Leu576del^, in our *KIT* mutant profile because segmental deletion is a more frequently detected KIT exon 11 mutation and associated with a worse clinical outcome after surgical resection than point mutation detected in advanced GIST [23]. In addition, we evaluated the inhibitory efficacy of TKIs at their clinically achievable Css, IR_Css_, which made the results can more readily be translated into clinical use. In current study, the IC_50_ of TKIs on the phosphorylation of exon 9 or 11/17 mutated KIT proteins was lowest for nilotinib followed by dasatinib, IM, SU, and sorafenib, which were largely comparable with the results in the study of Guo *et al.* [24]. However, considering the clinically achievable Css of each TKI, we found that nilotinib and sorafenib are more potent TKIs for IM/SU-resistant GISTs with secondary exon 17 mutation.

In several recent prospective and retrospective clinical studies as show in Table 1, nilotinib and sorafenib could achieve an overall DCR of 29–47% and 32%-42%, respectively, and a median PFS of 2.0–5.9 months and 4.9–5.2 months, respectively, as compared with that of 11% and 2.1 months in patients receiving best supportive care [25]–[32]. Moreover, a sorafenib analogue, regorafenib, has a broad spectrum of antitumor activity in preclinical and clinical benefit in IM/SU failure GISTs and recently been approved by the FDA as 3^rd^-line treatment for IM/SU-refractory GISTs [33]. Unfortunately, little information regarding the KIT genotype of IM/SU-resistant GIST was provided by these studies. As an example, in the series of Sawaki *et al.*, *KIT* genotyping of post-SU tumor tissue from two patients who achieved either partial response or disease control longer than 24 weeks after nilotinib, showed both tumors carried exon 11/17 double mutation [25]. In addition, the DCR at 24 weeks after nilotinib in patients receiving <6 weeks and >6 weeks of prior SU treatment was 33% and 18%, respectively. Considering the median PFS of IM-resistant GISTs harboring acquired secondary exon 17 mutation was noticeably shorter than that of patients with secondary exon 13/14 mutation, 2.3 months versus 7.8 months. Furthermore, Cauchi *et al.* found that the IM/SU-resistant GISTs of the only patient with prolonged disease stabilization (>12 moths) after 3^rd^-line nilotinib also harbored exon 11/17 double mutation [31]. In a phase II trial of 3^rd^-line dasatinib in IM/SU-resistant GISTs, Trent *et al.* found that patients with PDGFRA^Asp842Val^ mutated GISTs could achieve a better PFS than those with primary *KIT* mutated tumors [32]. Unfortunately, the genotyping of GIST resistant to IM and SU was not available in the report of 3^rd^-line sorafenib trials [26]–[28]. Taken together, these evidences support our findings that nilotinib may be a better agent for IM-resistant GIST with secondary exon 17 mutation than SU.

**Table 1:** Clinical outcomes of TKIs on IM/SU-resistant GIST.

Administration	Studies	N	SD>6 months	PFS (months)	OS (months)
Best supportive care	Italiano [26]	18	11%	2.1	2.4
Nilotinib	Italiano [26]	67	35%	4.1	11.8
	Cauchi [31]	13	7%	2.0	N.A.
	Montemurro [29]	52	47%	3	8.5
	Kim [30]	17	47%	5.9	18.5
	Sawaki [25]	35	29%	3.8	10.3
Sorafenib	Italiano [26]	55	42%	4.9	10.7
	Park [27]	31	36%	4.9	9.7
	Kindler [28]	32	32%	5.2	11.6
Dasatinib	Trent [32]	47	21%	3.8	10.3

S.D.: stable disease; PFS: progression-free survival; OS: overall survival; N.A: not available.

Furthermore, we also introduced molecular modeling to elucidate the interaction between TKIs and mutant KIT proteins. Previous study of Mol *et al.* first resolved the crystal structure of KIT and its phosphorylation status [34]. The molecular modeling of Mahadevan *et al.* showed that IM could not bind to Val654Ala mutant and explained the impact of KIT mutations on IM resistance [35]. In this study, we found that nilotinib had the best binding affinity for exon 11/17 (Fig. 6b), which is in consistent with our *in vitro* inhibitory efficacy study on KIT mutants. On the other hand, although the affinity of sorafenib to exon 11^Val555_Leu576del^/17^Asp820Gly^ mutant was intermediate in the molecular modeling study, the extremely high Css of sorafenib compared to other TKIs also contributes to effective inhibition on KIT exon 11/17 mutant. These findings pinpoint the importance of evaluating TKIs’ inhibitory efficacy on KIT at their Css rather than at equal concentration.

In conclusion, based on the clinical observation of potential risk of rapid progression in IM-refractory GISTs with secondary *KIT* exon 17 mutation receiving SU, we established a screening platform to evaluate and to compare the inhibitory activity of several commercially available TKIs on KIT activation at their clinically achievable Css for selecting optimal treatment for such patient population. The results indicate that nilotinib and sorafenib could more effectively inhibit the phosphorylation of KIT mutant with secondary exon 17 mutations in COS-1 model and the growth of GIST48 cells than IM and SU, which were supported by a molecular modeling study. An investigator-initiated, explorative randomization trials comparing either nilotinib or sorafenib against SU in IM-refractory, secondary *KIT* exon 17 mutation-enriched patient population is currently under discussion.

## Supporting Information

Figure S1 The comparisons of inhibitory effects of imatinib on KIT single mutants using western blotting analysis and Luminex assay. (a) COS-1 cells transfected with KIT single mutants were starved and treated with indicated doses of IM for 30 minutes. The total expression and degree of phosphorylation of KIT were determined by western blot analysis or Luminex assay (c). (b) Activation ratios of IM on KIT single mutations were determined by quantification of phosphorylated KIT/total KIT from the western blot in (a). The data are expressed as the mean ± SE of three independent experiments.
(TIFF)

Figure S2 Stereo views of SU and sorafenib binding to KIT showing key hydrogen bonds formed with A599 and R684 in different models.
(TIF)

Table S1 Primers used for mutagenesis of *KIT.* (a) Primers used for site directed mutagenesis of *KIT* exon 9^Ala502_Tyr503insAlaTyr^, 11^Val560Asp^, 11^Val555_Leu576del^, 13^Val654Ala^, 14^Thr670Ile^, and 17^Asp820Gly^, and 17^Asn822Lys^ mutants. (b) Primers used for slicing overlap extension of *KIT* exon 11^Val555_Leu576del^ mutants.
(DOC)
