# Supplementary figures and images for: Selecting Tyrosine Kinase Inhibitors for Gastrointestinal Stromal Tumor with Secondary KIT Activation-Loop Domain Mutations
Source: PLoS One. 2013 Jun 20;8(6):e65762. doi: 10.1371/journal.pone.0065762 (PMC3688691; doi:10.1371/journal.pone.0065762)

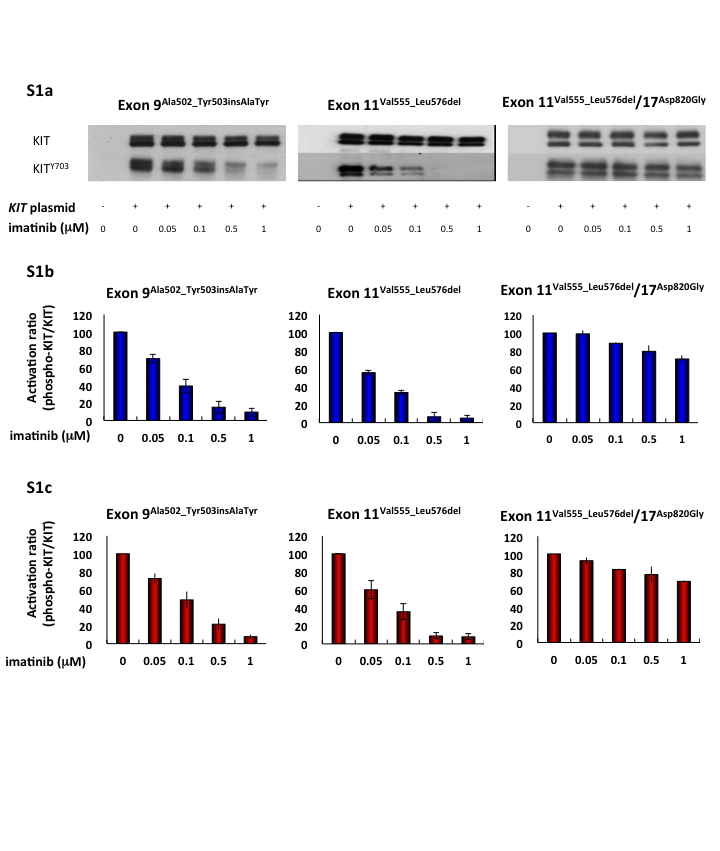

Supplement: Figure S1 — The comparisons of inhibitory effects of imatinib on KIT single mutants using western blotting analysis and Luminex assay. (a) COS-1 cells transfected with KIT single mutants were starved and treated with indicated doses of IM for 30 minutes. The total expression and degree of phosphorylation of KIT were determined by western blot analysis or Luminex assay (c). (b) Activation ratios of IM on KIT single mutations were determined by quantification of phosphorylated KIT/total KIT from the western blot in (a). The data are expressed as the mean ± SE of three independent experiments. (TIFF) [file pone.0065762.s001.tiff]

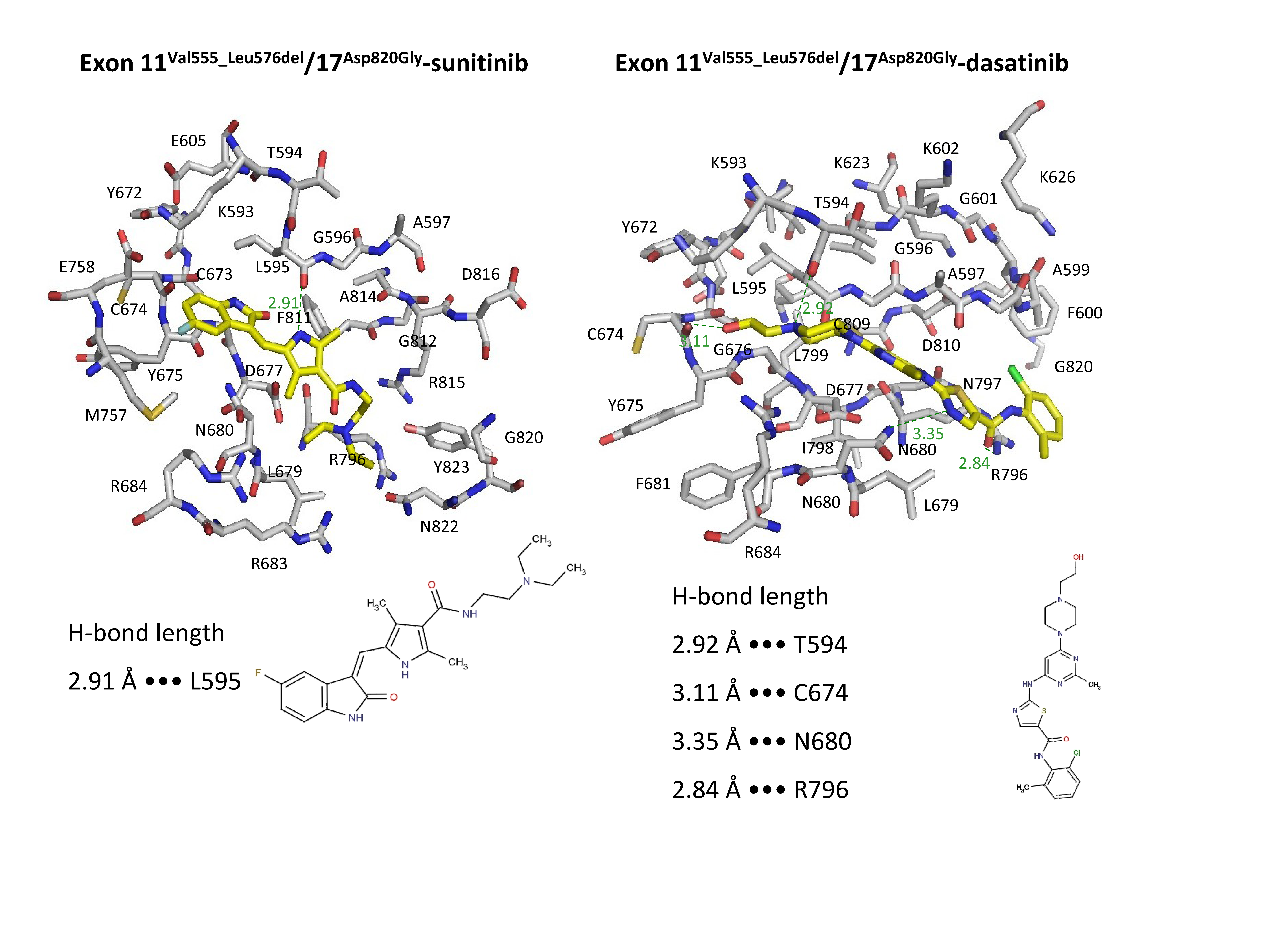

Supplement: Figure S2 — Stereo views of SU and sorafenib binding to KIT showing key hydrogen bonds formed with A599 and R684 in different models. (TIF) [file pone.0065762.s002.tif]
