# Supplementary material for: Selecting Tyrosine Kinase Inhibitors for Gastrointestinal Stromal Tumor with Secondary KIT Activation-Loop Domain Mutations
Source: PLoS One. 2013 Jun 20;8(6):e65762. doi: 10.1371/journal.pone.0065762 (PMC3688691; doi:10.1371/journal.pone.0065762)
Supplement: Table S1 — Primers used for mutagenesis of KIT. (a) Primers used for site directed mutagenesis of KIT exon 9Ala502_Tyr503insAlaTyr, 11Val560Asp, 11Val555_Leu576del, 13Val654Ala, 14Thr670Ile, and 17Asp820Gly, and 17Asn822Lys mutants. (b) Primers used for slicing overlap extension of KIT exon 11Val555_Leu576del mutants. (DOC) [file pone.0065762.s003.doc]

**S1a**

| Mutation site | Forward Primer | Reverse Primer |
| --- | --- | --- |
| Exon 9^Ala502_Tyr503insAlaTyr^ | TGGGCAAGACTTCTGCCTATGCCTATTTTAACTTTGCATTTAAAG | CTTTAAATGCAAAGTTAAAATAGGCATAGGCAGAAGTCTTGCCCA |
| Exon 11^Val560Asp^ | GAAGTACAGTGGAAGGTTGATGAGGAGATAAATGGAAAC | GTTTCCATTTATCTCCTCATCAACCTTCCACTGTACTTC |
| Exon 13^Val654Al^ | GGTAATCACATGAATATTGCGAATCTACTTGGAGCCTGC | GCAGGCTC CAAGTAGATT CGCAATATTCATGTGATTACC |
| Exon 14^Thr670Ile^ | GCACCATTGGAGGGCCCACCCTGGTCATTATAGAATATTGTTGC | GCAACAATATTCTATAATGACCAGGGTGGGCCCTCCAATGGTGC |
| Exon 17^Asp820Gly^ | GCCAGAGACATCAAGAATGGTTCTAATTATGTGG | CCACATAATTAGAAcCATTCTTGATGTCTCTGGC |
| Exon 17 ^Asn822Lys^ | GAGACATCAAGAATGATTCTAAaTATGTGGTTAAAGGAAACGC | GCGTTTCCTTTAACCACATAtTTAGAATCATTCTTGATGTCTC- |

**S1b**

|  | Exon 11^Val555_Leu576del^ |
| --- | --- |
| M1 | AAGCCTCTTCCCAAGGACTT |
| M2 | CCATTTGTGATCATAAGGTTCATACATGGGTTTCTG |
| M3 | CAGAAACCCATGTATGAACCTTATGATCACAAATGG |
| M4 | CGTTCTGTCAAATGGGCACT |
